# Distrontium oxalate tetra­hydroxidocuprate(II)

**DOI:** 10.1107/S241431462600043X

**Published:** 2026-01-20

**Authors:** Hibiki Kunisawa, Jun-ichi Yamaura, Toshihiro Nomura

**Affiliations:** a Department of Physics, Shizuoka University, Shizuoka 422-8529, Japan; b Institute for Solid State Physics, University of Tokyo, Kashiwa, Chiba 277-8581, Japan; Vienna University of Technology, Austria

**Keywords:** crystal structure, inorganic, oxalate

## Abstract

The crystal structure of distrontium oxalate tetra­hydroxidocuprate(II) or poly[tetra-μ-hydroxido-μ_6_-oxalato-copperdistrontium], Sr_2_(C_2_O_4_)[Cu(OH)_4_] or [Sr_2_Cu(C_2_O_4_)(OH)_4_]_*n*_, has been determined in the triclinic space group *P*1. The asymmetric unit contains one Sr, one Cu, two hydroxide groups, and half of an oxalate anion. By application of inversion symmetry, a square-planar {Cu(OH)_4_} unit and a complete oxalate anion are generated. The structure consists of a three-dimensional framework of edge-sharing {SrO_4_(OH)_4_} polyhedra decorated by {Cu(OH)_4_} units and oxalate groups. Only weak hydrogen bonds are observed within the framework.

## Abstract 2

Sr_2_Cu(OH)_4_(C_2_O_4_) is made up from edge-sharing {SrO_4_(OH)_4_} polyhedra decorated by {Cu(OH)_4_} units and oxalate groups.

## Graphical abstract

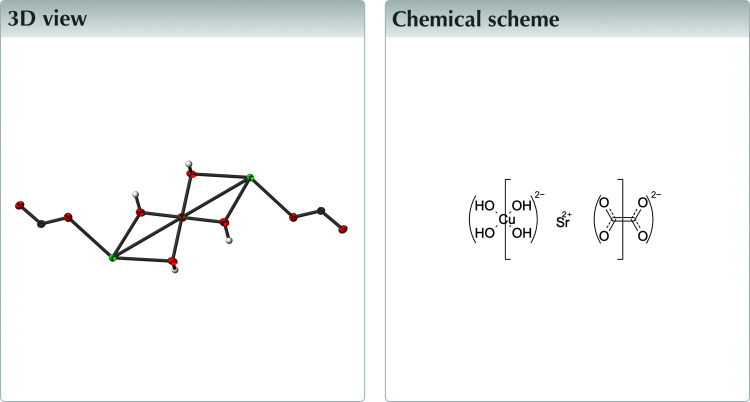

## Structure description

The title compound, Sr_2_(C_2_O_4_)[Cu(OH)_4_], was obtained serendipitously during attempts to synthesize SrCu_2_(BO_3_)_2_ (Kageyama *et al.*, 1999 ▸) under hydro­thermal conditions. Although numerous crystal structures containing Cu^II^ and oxalato ligands or oxalate anions have been reported, the combination with alkaline-earth ions is surprisingly rare. According to the Inorganic Crystal Structure Database (ICSD; version 2025–1; Zagorac *et al.*, 2019 ▸), only a few related compounds such as Sr_2_(Cu(C_2_O_4_)_3_)(H_2_O)_7_ (Insausti *et al.*, 1994 ▸) and BaCu(C_2_O_4_)_2_·6H_2_O (Hallock *et al.*, 1990 ▸; Bouayad *et al.*, 1995 ▸; Kasthuri *et al.*, 1996 ▸; Nenwa *et al.*, 2008 ▸) have been reported. Insausti and coworkers also reported the thermal analysis of CaCu(C_2_O_4_)_2_·2H_2_O and SrCu(C_2_O_4_)_2_·4H_2_O, yet the crystal structures of these compounds have not been determined (Insausti *et al.*, 1993 ▸). Here, we describe the crystal structure of Sr_2_(C_2_O_4_)[Cu(OH)_4_].

**Figure 1:**
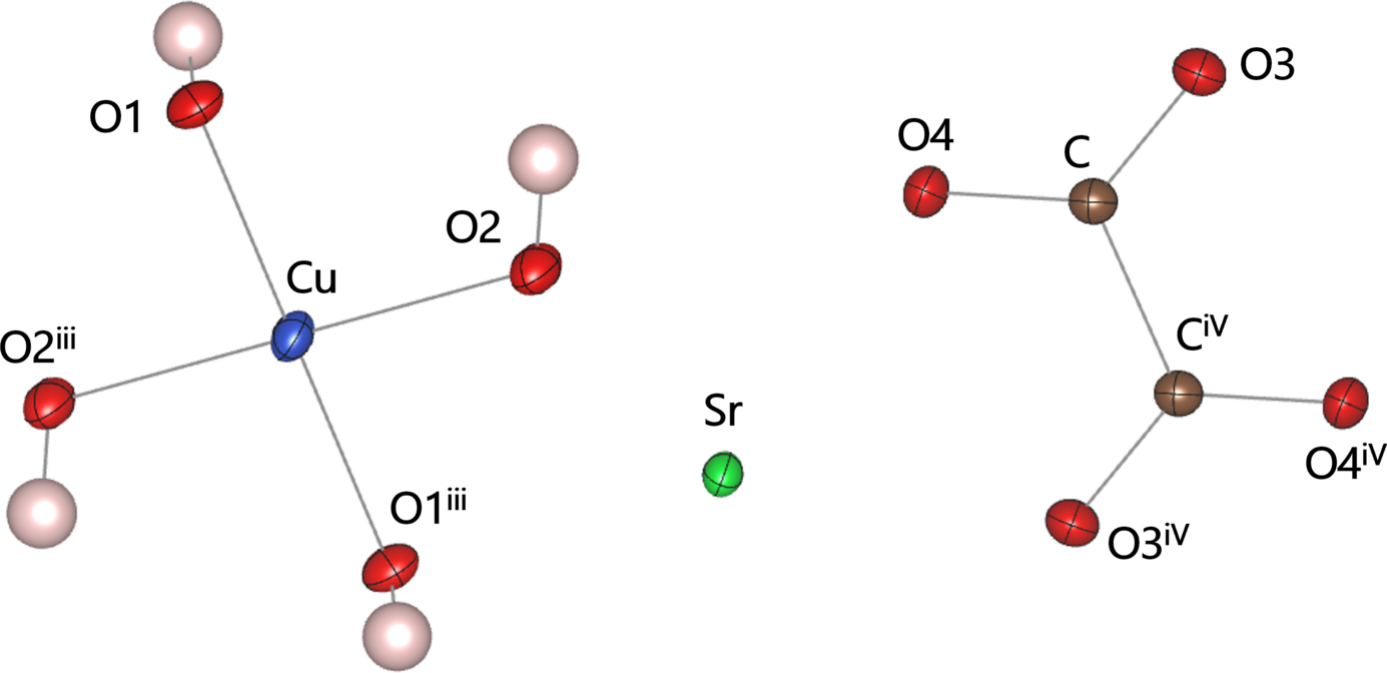
The asymmetric unit of the title compound expanded to visualize the complete {Cu(OH)_4_}^2–^ unit and the oxalate anion. Displacement ellipsoids are drawn at the 50% probability level. [Symmetry codes: (iii) −*x* + 1, −*y*, −*z* + 1, (iv) −*x*, −*y* + 1, −*z* + 2.]

**Figure 3:**
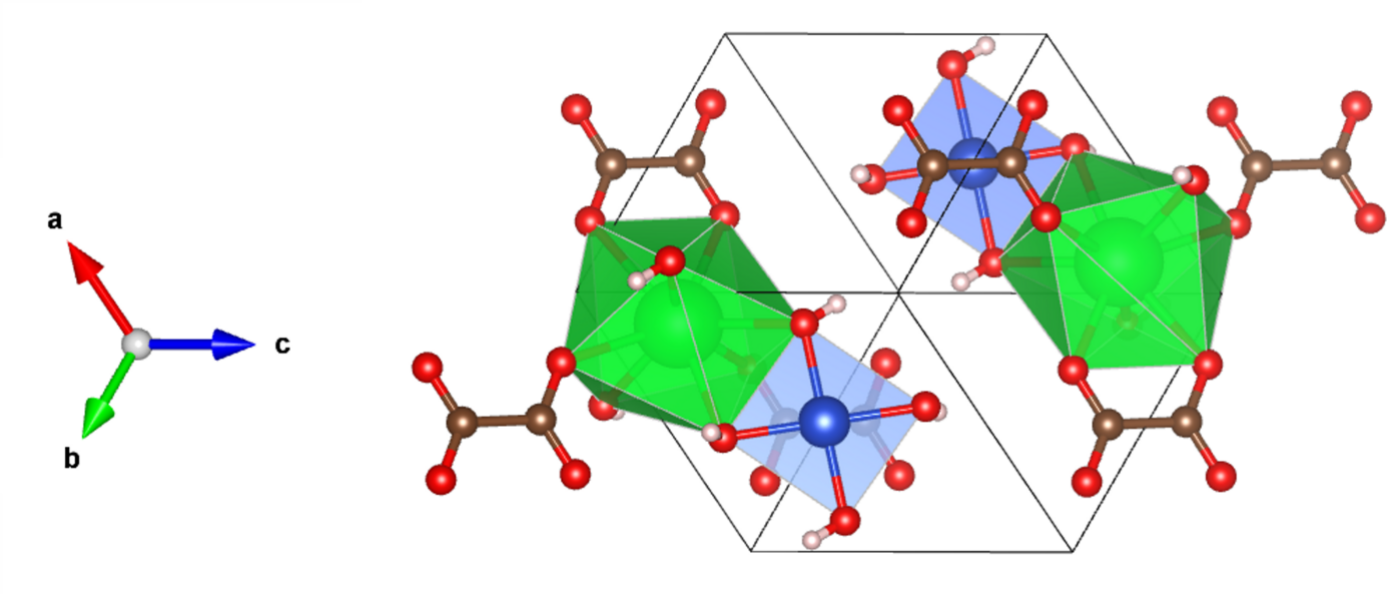
The expanded unit cell of the title compound viewed along the [111] direction; color codes are as in Fig. 2 ▸.

The crystal structure of Sr_2_(C_2_O_4_)[Cu(OH)_4_] consists of a three-dimensional framework built from Sr^II^ cations coordinated by {Cu(OH)_4_}^2–^ and oxalate (C_2_O_4_)^2–^ units (Figs. 1 ▸ and 2 ▸). The asymmetric unit comprises one Sr^II^, one Cu^II^, two (OH) groups, and half of an oxalate anion. By application of inversion symmetry, a {Cu(OH)_4_} square-planar unit and the full oxalate anion are generated. The coordination environment around Sr is an {SrO_4_(OH)_4_} polyhedron, which resembles a square anti­prism but is significantly distorted in the triclinic lattice. Each oxalate anion bonds to six Sr^II^ cations, with four bridging and two chelating modes. The {Cu(OH)_4_} units are oriented nearly perpendicular to the crystallographic [111] direction (Fig. 3 ▸). The OH^−^ groups of the {Cu(OH)_4_} unit do not form obvious hydrogen bonds with the surrounding oxygen atoms of the oxalate anions. The O1⋯O4′ and O2⋯O3′ distances are around 3.0 Å, however, the O—H⋯O angles are strongly bent from 180° (104 and 121°, respectively), indicating that these hydrogen bonds are rather weak.

## Synthesis and crystallization

Sr(OH)_2_·8H_2_O (1.4 g), Cu(OH)_2_ (0.1 g), acetyl­acetone (C_5_H_8_O_2_, 0.2 ml), H_3_BO_3_ (0.03 g), and distilled water (10 ml) were placed in a Teflon-lined stainless-steel autoclave and heated at 473 K for 24 h. All reagents were purchased from FUJIFILM Wako and used without further purification. The oxalate ions are likely generated through the oxidative decomposition of acetyl­acetone under the alkaline reaction conditions. Violet, rhombic plates were obtained, and a single crystal was selected for X-ray diffraction at room temperature.

## Refinement

Crystal data, data collection, and structure refinement details are summarized in Table 1 ▸. H atoms were located from difference syntheses and were refined using a riding model (AFIX 147 instruction; Sheldrick, 2015*b* ▸).

## Supplementary Material

Crystal structure: contains datablock(s) I. DOI: 10.1107/S241431462600043X/wm4243sup1.cif

Structure factors: contains datablock(s) I. DOI: 10.1107/S241431462600043X/wm4243Isup2.hkl

CCDC reference: 2523251

Additional supporting information:  crystallographic information; 3D view; checkCIF report

## Figures and Tables

**Figure 2:**
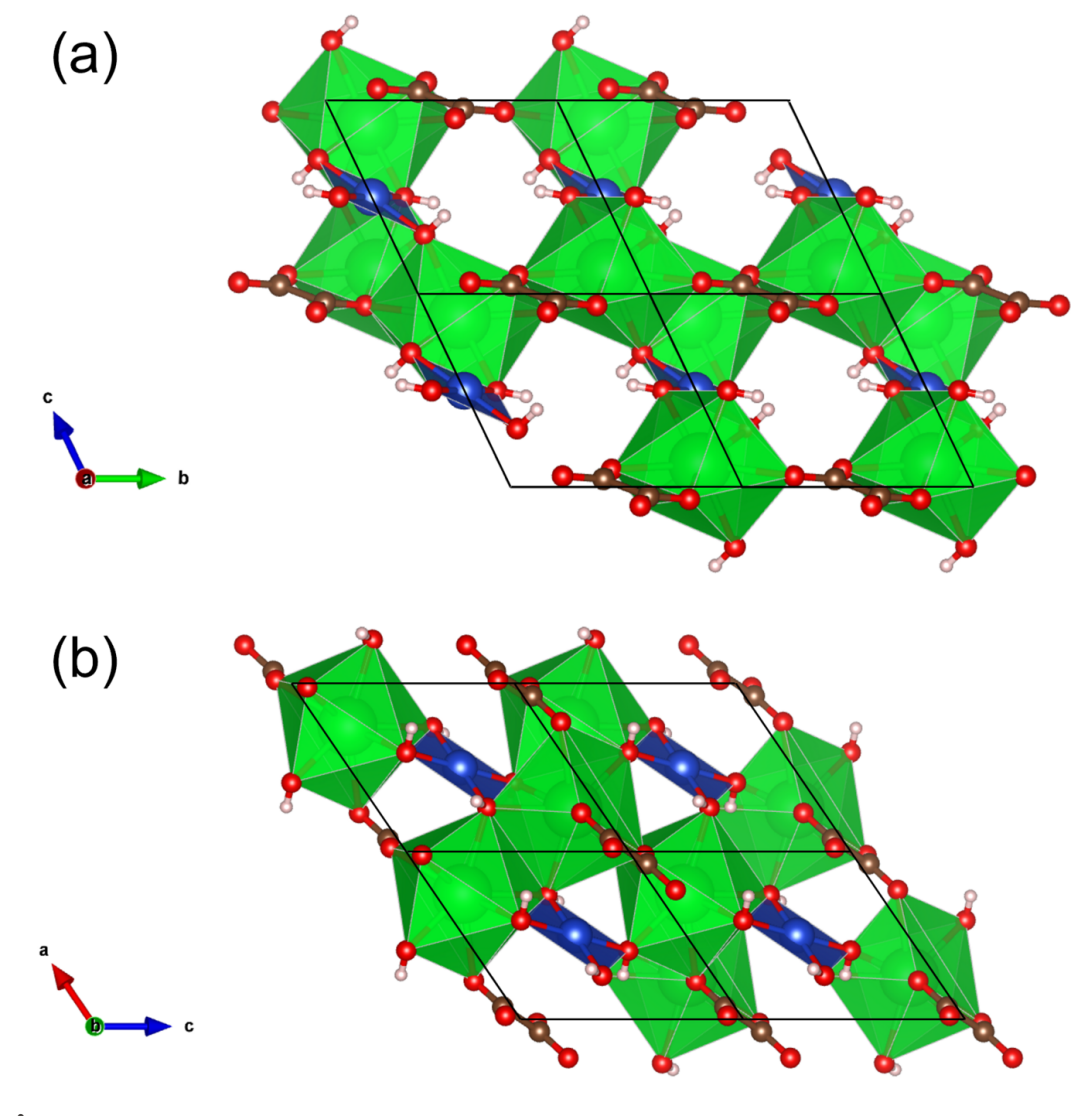
Three-dimensional framework of the crystal structure with polyhedral representation of the {SrO_4_(OH)_4_} (green) and {Cu(OH)_4_} (blue) building units, as viewed along the (*a*) *a* and (*b*) *b* axes.

**Table 1:** Experimental details

Crystal data	
Chemical formula	[Sr_2_Cu(C_2_O_4_)(OH)_4_]
*M*_r_	394.84
Crystal system, space group	Triclinic, *P* 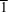
Temperature (K)	293
*a*, *b*, *c* (Å)	6.0754 (3), 6.5442 (3), 6.5466 (2)
α, β, γ (°)	103.712 (3), 117.235 (4), 106.601 (4)
*V* (Å^3^)	200.06 (2)
*Z*	1
Radiation type	Cu *K*α
μ (mm^−1^)	20.54
Crystal size (mm)	0.08 × 0.06 × 0.01

Data collection	
Diffractometer	XtaLAB Synergy R, HyPix
Absorption correction	Multi-scan (*CrysAlis PRO*; Rigaku OD, 2023 ▸)
*T*_min_, *T*_max_	0.705, 1.000
No. of measured, independent and observed [*I* > 2σ(*I*)] reflections	1612, 785, 774
*R*_int_	0.019
(sin θ/λ)_max_ (Å^−1^)	0.631

Refinement	
*R*[*F*^2^ > 2σ(*F*^2^)], *wR*(*F*^2^), *S*	0.018, 0.049, 1.07
No. of reflections	785
No. of parameters	64
H-atom treatment	H-atom parameters constrained
Δρ_max_, Δρ_min_ (e Å^−3^)	0.49, −0.48

Computer programs: *CrysAlis PRO* (Rigaku OD, 2023 ▸), *SHELXT* (Sheldrick, 2015*a* ▸), *SHELXL* (Sheldrick, 2015*b* ▸), *VESTA 3* (Momma & Izumi, 2011 ▸) and *publCIF* (Westrip, 2010 ▸).

## full crystallographic data

### Poly[tetra-µ-hydroxido-µ6-oxalato-copperdistrontium] Crystal data

**Table:**

[CuSr_2_(C_2_O_4_)(OH)_4_]	*Z* = 1
*M**_r_* = 394.84	*F*(000) = 185
Triclinic, *P*1	*D*_x_ = 3.277 Mg m^−^^3^
*a* = 6.0754 (3) Å	Cu *K*α radiation, λ = 1.54184 Å
*b* = 6.5442 (3) Å	Cell parameters from 1429 reflections
*c* = 6.5466 (2) Å	θ = 7.8–75.7°
α = 103.712 (3)°	µ = 20.54 mm^−^^1^
β = 117.235 (4)°	*T* = 293 K
γ = 106.601 (4)°	Plate, translucent, violet
*V* = 200.06 (2) Å^3^	0.08 × 0.06 × 0.01 mm

### Poly[tetra-µ-hydroxido-µ6-oxalato-copperdistrontium] Data collection

**Table:**

XtaLAB Synergy R, HyPix diffractometer	785 independent reflections
Radiation source: Rotating-anode X-ray tube	774 reflections with *I* > 2σ(*I*)
Detector resolution: 10.0000 pixels mm^-1^	*R*_int_ = 0.019
ω scans	θ_max_ = 76.6°, θ_min_ = 7.8°
Absorption correction: multi-scan (CrysAlisPro; Rigaku OD, 2023)	*h* = −7→7
*T*_min_ = 0.705, *T*_max_ = 1.000	*k* = −4→8
1612 measured reflections	*l* = −8→8

### Poly[tetra-µ-hydroxido-µ6-oxalato-copperdistrontium] Refinement

**Table:**

Refinement on *F*^2^	Hydrogen site location: inferred from neighbouring sites
Least-squares matrix: full	H-atom parameters constrained
*R*[*F*^2^ > 2σ(*F*^2^)] = 0.018	*w* = 1/[σ^2^(*F*_o_^2^) + (0.0306*P*)^2^ + 0.1268*P*] where *P* = (*F*_o_^2^ + 2*F*_c_^2^)/3
*wR*(*F*^2^) = 0.049	(Δ/σ)_max_ < 0.001
*S* = 1.07	Δρ_max_ = 0.49 e Å^−^^3^
785 reflections	Δρ_min_ = −0.48 e Å^−^^3^
64 parameters	Extinction correction: SHELXL2019/2 (Sheldrick 2015b), Fc^*^=kFc[1+0.001xFc^2^λ^3^/sin(2θ)]^-1/4^
0 restraints	Extinction coefficient: 0.0151 (9)

### Poly[tetra-µ-hydroxido-µ6-oxalato-copperdistrontium] Special details

**Table:**

Geometry. All esds (except the esd in the dihedral angle between two l.s. planes) are estimated using the full covariance matrix. The cell esds are taken into account individually in the estimation of esds in distances, angles and torsion angles; correlations between esds in cell parameters are only used when they are defined by crystal symmetry. An approximate (isotropic) treatment of cell esds is used for estimating esds involving l.s. planes.
Refinement. Hydrogen atoms were located from difference syntheses. All O—H hydrogen atoms were refined using the AFIX 147 riding model (Sheldrick, 2015*b*).

### Poly[tetra-µ-hydroxido-µ6-oxalato-copperdistrontium] Fractional atomic coordinates and isotropic or equivalent isotropic displacement parameters (Å^2^)

**Table:**

	*x*	*y*	*z*	*U*_iso_*/*U*_eq_	
Sr	0.25216 (5)	0.13033 (4)	0.87805 (4)	0.01415 (14)	
Cu	0.500000	0.000000	0.500000	0.01451 (17)	
C	0.0730 (5)	0.5793 (4)	0.9551 (5)	0.0137 (5)	
O1	0.5900 (4)	0.1511 (4)	0.3047 (4)	0.0187 (4)	
O2	0.2604 (4)	0.1402 (4)	0.4994 (4)	0.0194 (4)	
O3	0.0218 (5)	0.7498 (4)	0.9402 (4)	0.0228 (4)	
O4	0.2296 (5)	0.5243 (4)	0.9062 (5)	0.0234 (5)	
H1	0.733912	0.275791	0.402865	0.028*	
H2	0.299239	0.256363	0.471123	0.029*	

### Poly[tetra-µ-hydroxido-µ6-oxalato-copperdistrontium] Atomic displacement parameters (Å^2^)

**Table:**

	*U*^11^	*U*^22^	*U*^33^	*U*^12^	*U*^13^	*U*^23^
Sr	0.01757 (18)	0.01766 (18)	0.01888 (19)	0.01191 (12)	0.01421 (14)	0.01195 (13)
Cu	0.0152 (3)	0.0194 (3)	0.0155 (3)	0.0094 (2)	0.0114 (3)	0.0095 (2)
C	0.0144 (12)	0.0129 (12)	0.0151 (13)	0.0064 (10)	0.0088 (11)	0.0070 (10)
O1	0.0232 (10)	0.0207 (10)	0.0206 (10)	0.0102 (8)	0.0162 (9)	0.0128 (8)
O2	0.0220 (10)	0.0264 (11)	0.0246 (11)	0.0154 (9)	0.0175 (9)	0.0181 (9)
O3	0.0315 (11)	0.0201 (10)	0.0370 (12)	0.0174 (9)	0.0268 (10)	0.0193 (9)
O4	0.0291 (11)	0.0220 (10)	0.0416 (13)	0.0169 (9)	0.0297 (11)	0.0197 (10)

### Poly[tetra-µ-hydroxido-µ6-oxalato-copperdistrontium] Geometric parameters (Å, º)

**Table:**

Sr—O2	2.518 (2)	Sr—Sr^ii^	3.9910 (5)
Sr—O1^i^	2.539 (2)	Cu—O2^iii^	1.9287 (18)
Sr—O2^ii^	2.583 (2)	Cu—O2	1.9287 (18)
Sr—O4	2.588 (2)	Cu—O1^iii^	1.9603 (19)
Sr—O1^iii^	2.600 (2)	Cu—O1	1.9603 (19)
Sr—O3^iv^	2.630 (2)	C—O4	1.248 (3)
Sr—O3^v^	2.708 (2)	C—O3	1.254 (3)
Sr—O4^vi^	2.714 (2)	C—C^iv^	1.565 (5)
Sr—Cu	3.5020 (2)	O1—H1	0.8200
Sr—Cu^vii^	3.7623 (3)	O2—H2	0.8200
Sr—Sr^viii^	3.7886 (4)		
			
O2—Sr—O1^i^	135.38 (6)	O4—Sr—Sr^ii^	88.07 (5)
O2—Sr—O2^ii^	77.03 (7)	O1^iii^—Sr—Sr^ii^	74.17 (5)
O1^i^—Sr—O2^ii^	137.35 (6)	O3^iv^—Sr—Sr^ii^	106.34 (5)
O2—Sr—O4	80.99 (6)	O3^v^—Sr—Sr^ii^	99.03 (5)
O1^i^—Sr—O4	113.62 (7)	O4^vi^—Sr—Sr^ii^	115.39 (5)
O2^ii^—Sr—O4	95.78 (7)	Cu—Sr—Sr^ii^	59.857 (6)
O2—Sr—O1^iii^	63.16 (6)	Cu^vii^—Sr—Sr^ii^	53.604 (6)
O1^i^—Sr—O1^iii^	85.00 (7)	Sr^viii^—Sr—Sr^ii^	115.634 (11)
O2^ii^—Sr—O1^iii^	91.06 (6)	O2^iii^—Cu—O2	180.0
O4—Sr—O1^iii^	140.92 (6)	O2^iii^—Cu—O1^iii^	92.84 (8)
O2—Sr—O3^iv^	132.05 (6)	O2—Cu—O1^iii^	87.16 (8)
O1^i^—Sr—O3^iv^	89.39 (7)	O2^iii^—Cu—O1	87.16 (8)
O2^ii^—Sr—O3^iv^	77.40 (7)	O2—Cu—O1	92.84 (8)
O4—Sr—O3^iv^	62.15 (6)	O1^iii^—Cu—O1	180.00 (6)
O1^iii^—Sr—O3^iv^	156.01 (6)	O2^iii^—Cu—Sr	135.56 (6)
O2—Sr—O3^v^	128.48 (7)	O2—Cu—Sr	44.44 (6)
O1^i^—Sr—O3^v^	68.85 (7)	O1^iii^—Cu—Sr	47.09 (6)
O2^ii^—Sr—O3^v^	68.65 (7)	O1—Cu—Sr	132.91 (6)
O4—Sr—O3^v^	138.09 (6)	O2^iii^—Cu—Sr^iii^	44.44 (6)
O1^iii^—Sr—O3^v^	79.93 (6)	O2—Cu—Sr^iii^	135.56 (6)
O3^iv^—Sr—O3^v^	76.30 (7)	O1^iii^—Cu—Sr^iii^	132.91 (6)
O2—Sr—O4^vi^	76.83 (7)	O1—Cu—Sr^iii^	47.09 (6)
O1^i^—Sr—O4^vi^	69.84 (7)	Sr—Cu—Sr^iii^	180.0
O2^ii^—Sr—O4^vi^	152.23 (6)	O2^iii^—Cu—Sr^ii^	140.54 (6)
O4—Sr—O4^vi^	71.00 (7)	O2—Cu—Sr^ii^	39.46 (6)
O1^iii^—Sr—O4^vi^	85.41 (6)	O1^iii^—Cu—Sr^ii^	86.22 (6)
O3^iv^—Sr—O4^vi^	114.46 (7)	O1—Cu—Sr^ii^	93.78 (6)
O3^v^—Sr—O4^vi^	137.07 (7)	Sr—Cu—Sr^ii^	66.539 (7)
O2—Sr—Cu	32.44 (4)	Sr^iii^—Cu—Sr^ii^	113.461 (8)
O1^i^—Sr—Cu	106.01 (4)	O2^iii^—Cu—Sr^ix^	39.46 (6)
O2^ii^—Sr—Cu	92.68 (4)	O2—Cu—Sr^ix^	140.54 (6)
O4—Sr—Cu	107.57 (5)	O1^iii^—Cu—Sr^ix^	93.78 (6)
O1^iii^—Sr—Cu	33.52 (4)	O1—Cu—Sr^ix^	86.22 (6)
O3^iv^—Sr—Cu	164.31 (5)	Sr—Cu—Sr^ix^	113.461 (7)
O3^v^—Sr—Cu	111.68 (4)	Sr^iii^—Cu—Sr^ix^	66.539 (8)
O4^vi^—Sr—Cu	69.58 (5)	Sr^ii^—Cu—Sr^ix^	180.0
O2—Sr—Cu^vii^	87.84 (5)	O4—C—O3	126.4 (2)
O1^i^—Sr—Cu^vii^	136.43 (5)	O4—C—C^iv^	116.8 (3)
O2^ii^—Sr—Cu^vii^	28.33 (4)	O3—C—C^iv^	116.8 (3)
O4—Sr—Cu^vii^	71.54 (5)	Cu—O1—Sr^x^	127.46 (10)
O1^iii^—Sr—Cu^vii^	119.12 (5)	Cu—O1—Sr^iii^	99.39 (8)
O3^iv^—Sr—Cu^vii^	53.30 (5)	Sr^x^—O1—Sr^iii^	95.00 (7)
O3^v^—Sr—Cu^vii^	79.79 (5)	Cu—O1—H1	109.5
O4^vi^—Sr—Cu^vii^	141.23 (4)	Sr^x^—O1—H1	115.8
Cu—Sr—Cu^vii^	113.461 (7)	Sr^iii^—O1—H1	103.2
O2—Sr—Sr^viii^	99.63 (4)	Cu—O2—Sr	103.13 (8)
O1^i^—Sr—Sr^viii^	43.12 (4)	Cu—O2—Sr^ii^	112.21 (9)
O2^ii^—Sr—Sr^viii^	120.37 (4)	Sr—O2—Sr^ii^	102.97 (7)
O4—Sr—Sr^viii^	143.24 (5)	Cu—O2—H2	109.5
O1^iii^—Sr—Sr^viii^	41.88 (5)	Sr—O2—H2	126.4
O3^iv^—Sr—Sr^viii^	128.30 (5)	Sr^ii^—O2—H2	102.5
O3^v^—Sr—Sr^viii^	68.79 (5)	C—O3—Sr^iv^	120.51 (17)
O4^vi^—Sr—Sr^viii^	73.39 (4)	C—O3—Sr^xi^	133.28 (17)
Cu—Sr—Sr^viii^	67.216 (6)	Sr^iv^—O3—Sr^xi^	103.70 (7)
Cu^vii^—Sr—Sr^viii^	145.022 (10)	C—O4—Sr	122.20 (17)
O2—Sr—Sr^ii^	39.09 (5)	C—O4—Sr^vi^	117.75 (17)
O1^i^—Sr—Sr^ii^	157.66 (5)	Sr—O4—Sr^vi^	109.00 (7)
O2^ii^—Sr—Sr^ii^	37.93 (4)		
			
O4—C—O3—Sr^iv^	−170.3 (2)	O3—C—O4—Sr	−170.1 (2)
C^iv^—C—O3—Sr^iv^	9.5 (4)	C^iv^—C—O4—Sr	10.2 (4)
O4—C—O3—Sr^xi^	−11.5 (5)	O3—C—O4—Sr^vi^	49.9 (4)
C^iv^—C—O3—Sr^xi^	168.3 (2)	C^iv^—C—O4—Sr^vi^	−129.9 (3)

Symmetry codes: (i) *x*, *y*, *z*+1; (ii) −*x*, −*y*, −*z*+1; (iii) −*x*+1, −*y*, −*z*+1; (iv) −*x*, −*y*+1, −*z*+2; (v) *x*, *y*−1, *z*; (vi) −*x*+1, −*y*+1, −*z*+2; (vii) *x*−1, *y*, *z*; (viii) −*x*+1, −*y*, −*z*+2; (ix) *x*+1, *y*, *z*; (x) *x*, *y*, *z*−1; (xi) *x*, *y*+1, *z*.
